# Bilirubin-Driven Phenotype Identifies Higher Mortality Risk in Severe Alcohol-Associated Hepatitis

**DOI:** 10.1016/j.gastha.2026.100982

**Published:** 2026-04-24

**Authors:** Tewodros T. Ayele, Tomohiro Tanaka

**Affiliations:** 1 Division of Gastroenterology and Hepatology, Department of Internal Medicine, University of Iowa Hospitals and Clinics, Iowa City, Iowa; 2 Department of Health Management and Policy, University of Iowa College of Public Health, Iowa City, Iowa; 3 Center for Access & Delivery Research and Evaluation (CADRE), Iowa City VA Health Care System, Iowa City, Iowa

**Keywords:** Severe Alcohol-Associated Hepatitis, Prognosis, Prothrombin Time, 90-Day Mortality, Cluster Analysis, Maddrey’s Discriminant Function (MDF)

## Abstract

**Background and Aims:**

Among patients with severe alcohol-associated hepatitis (AH), the prognostic relevance of the dominant component of Maddrey’s Discriminant Function (MDF) is unclear.

**Methods:**

We conducted a retrospective cohort study of adults with AH treated with corticosteroids at a US academic center (January 2016–December 2024). Using K-means clustering of the bilirubin and prothrombin time (PT) components of MDF, patients were classified into “PT-driven” vs “bilirubin-driven” phenotypes. The primary outcome was 90-day all-cause mortality. Inverse probability of treatment weighting-weighted Cox proportional hazards models were performed under an intention-to-treat framework, in which patients were not censored at liver transplantation. Secondary analyses used Fine-Gray competing risk models with liver transplantation as a competing event.

**Results:**

Among 212 patients, 56 (26.4%) died within 90 days. Despite a similar Model for End-Stage Liver Disease, the bilirubin-driven group (n = 100) had significantly lower MDF scores and less hepatic encephalopathy compared to the PT-driven group (n = 112). Nevertheless, in the inverse probability of treatment weighting-weighted Cox proportional hazards model, the bilirubin-driven phenotype was associated with higher 90-day mortality (hazard ratio, 2.06; 95% confidence interval, 1.05–4.04). Findings were consistent in the Fine-Gray competing risks model (subdistribution hazard ratio, 2.11; 95% confidence interval, 1.03–4.35).

**Conclusion:**

A bilirubin-dominant phenotype in severe AH was associated with significantly higher 90-day mortality, highlighting disease heterogeneity and suggesting potential value for risk stratification and for guiding future research on individualized care and optimal timing of liver transplant evaluation.

## Graphical abstract

**Figure:**
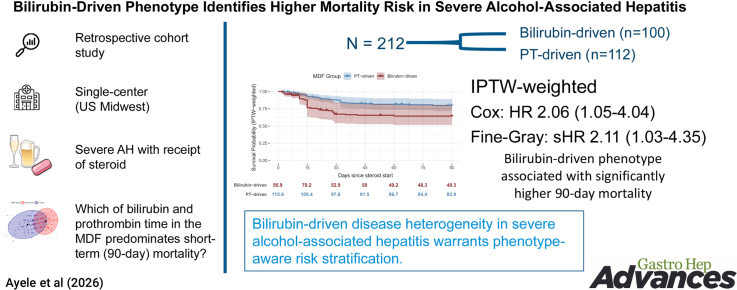

## Introduction

Alcohol-associated hepatitis (AH) is an acute inflammatory syndrome occurring in individuals with chronic and excessive alcohol use and carries a high short-term mortality.^1^, ^2^, ^3^ AH is primarily diagnosed clinically rather than by liver biopsy.^4^ According to the National Institute on Alcohol Abuse and Alcoholism, diagnostic criteria include heavy alcohol consumption for ≥6 months, <60 days of abstinence before the onset of jaundice, elevated serum bilirubin >3 mg/dL (the onset of which should be within the prior 8 weeks), aspartate aminotransferase (AST) >50 international unit (IU)/L with an AST/alanine aminotransferase (ALT) ratio >1.5, and transaminases typically <400 IU/L.^4^

Corticosteroids remain the only medical therapy widely accepted to improve short-term survival in severe AH.^2^^,^^3^ Eligibility for corticosteroid treatment is commonly assessed using static prognostic scores,^5^ most traditionally Maddrey’s Discriminant Function (MDF),^6^ and more recently, the Model for End-Stage Liver Disease (MELD) due to the increasingly recognized limitations of MDF in prognostic accuracy.^7^, ^8^, ^9^, ^10^ Treatment response is evaluated using the Lille model, which is a dynamic score based on the change in selected clinical parameters after 7 (or 4) days of corticosteroids.^11^^,^^12^

Both MDF and MELD incorporate multiple laboratory variables, including bilirubin and prothrombin time (PT), yet the individual prognostic contribution of each remains unclear. In other words, it is not known whether patients whose disease is driven more by bilirubin than PT, or vice versa, have different outcomes. Current models treat AH as a single entity and do not account for biologically distinct phenotypes. Furthermore, no study has evaluated whether the dominant component of MDF (bilirubin vs PT) independently predicts short-term outcomes. One study reported that baseline PT predicts steroid nonresponse,^13^ but it used the Lille score as the outcome, and because Lille includes baseline PT as its only PT-related component, this finding may reflect mathematical coupling rather than a true biological effect.

To address this gap, we applied a clustering approach (K-means clustering) using MDF components (ie, bilirubin and PT) to identify distinct clinical phenotypes and evaluated their association with short-term (90-day) all-cause mortality.

## Methods

This study was approved by the University of Iowa Institutional Review Board, which waived the requirement for informed consent given the retrospective design (IRB ID: 202507540).

### Study Design and Population

We conducted a retrospective cohort study of adult patients with severe AH who were treated with corticosteroids at the University of Iowa Hospitals and Clinics between January 2016 and December 2024. This study adhered to the Strengthening the Reporting of Observational Studies in Epidemiology guidelines.^14^ Patients were identified through structured chart review and included if they met the National Institute on Alcohol Abuse and Alcoholism clinical criteria^4^: (1) onset of jaundice within the prior 8 weeks; (2) total serum bilirubin >3.0 mg/dL; (3) ongoing consumption of >40 g/day (women) or >60 g/day (men) of alcohol for ≥6 months, with <60 days of abstinence before jaundice onset; and (4) AST >50 IU/L, AST/ALT ratio >1.5, and both AST and ALT <400 IU/L. We excluded patients with missing AST or international normalized ratio values, alternative etiologies of liver disease, or insufficient data to ascertain outcomes.

To classify patients based on the dominant biochemical driver of disease severity, we calculated the MDF^6^ using the standard formula: 4.6 × (PT − control PT) + bilirubin. To differentiate patterns in the contribution of PT and bilirubin to the MDF score, we standardized both components (rescaled to mean zero and standard deviation 1) to ensure comparability of scale and variance.

To classify patients by the dominant driver of their MDF score, we applied K-means clustering^15^ to the standardized PT-derived and bilirubin-derived MDF components. The optimal number of clusters was determined using average silhouette width^16^ (k = 2 through 7), which supported k = 2, yielding PT-driven and bilirubin-driven subgroups (see Supplementary Appendix). This clustering approach, adapted from prior work on MELD subtype classification,^17^ partitioned patients into 2 groups by minimizing within-cluster variance in a 2-dimensional space defined by the standardized PT- and bilirubin-derived MDF components, with k = 2 selected based on average silhouette width as described above. The resulting phenotypes were labeled “bilirubin-driven” and “PT-driven” based on the dominant contributor to the MDF score. To evaluate the separation and interpretability of the clusters, we visualized the data in 2-dimensional space.

We described the clinical and demographic characteristics of the study sample, including potential confounders.

### Statistical Framework

The primary outcome was 90-day all-cause mortality from the date of corticosteroid initiation. Patients were followed until death, censoring at 90 days, or last known follow-up. In the primary analysis, we used an intention-to-treat framework: patients who underwent liver transplantation (LT) remained in the risk set and were not censored. In secondary analysis, we constructed competing risk models to account for LT as a competing risk. All analyses were conducted using R version 4.4.1 (R Foundation for Statistical Computing, Vienna, Austria), and statistical significance was defined as a 2-sided *P* value <.05.

### Survival Analysis

Inverse probability of treatment weighting (IPTW)^18^^,^^19^ was used to balance baseline characteristics between the bilirubin-driven and PT-driven groups. Propensity scores were estimated using logistic regression including the following covariates: MDF score (continuous), age, sex, race, insurance type, intensive care unit stay, gastrointestinal bleeding, hepatic encephalopathy (HE), sepsis, baseline laboratory values (AST, albumin, sodium, creatinine), and corticosteroid regimen. Stabilized weights targeting the average treatment effect were applied, and extreme weights were trimmed at the 99th percentile. Covariate balance before and after weighting was assessed using standardized mean differences and visualized with a Love plot.

Survival differences were assessed using IPTW-weighted Kaplan-Meier (KM) analysis. KM curves were constructed to compare 90-day survival between the bilirubin-driven and PT-driven groups. To estimate the marginalized association between MDF phenotype and 90-day mortality, we used Cox proportional hazards (PH) models, treating cluster assignment (bilirubin-driven vs PT-driven) as the primary exposure. This was conducted as an intention-to-treat analysis, with LT not treated as a censoring event. A hazard ratio (HR) with 95% confidence intervals (CIs) was reported. The PH assumption was evaluated using Schoenfeld residuals. To account for LT as a competing event, we also constructed Fine-Gray hazard models using the *timereg* package in R.^20^ The model estimated the subdistribution HR (sHR) for 90-day mortality.

### Lille Score and Steroid Discontinuation

We evaluated response to corticosteroids using the Lille score,^11^ calculated on day 7 in patients who remained alive and had lab data. Missing values (bilirubin, creatinine, albumin), which accounted for <10% of all observations, were imputed using predictive mean matching.^21^ Lille scores were computed using standard inputs and compared between MDF phenotypes using *t*-tests and Wilcoxon tests. We also compared steroid discontinuation at discharge, defined as absence of outpatient prednisone/prednisolone, between groups using chi-square tests.

## Results

A total of 212 patients were included in the final analysis (Table 1). The mean age was 45.5 years (standard deviation, 11.8), and 73% were male. Over the 90-day observation period following corticosteroid initiation, 56 patients (26.4%) died and 12 patients (6%) underwent LT.

**Table 1:** Patient Characteristics

Characteristic	Bilirubin-driven (n = 100)	PT-driven (n = 112)	*P* value
Demographics			
Age at admission, mean (SD)	44.91 (10.55)	47.40 (11.24)	.099
Female, n (%)	33 (33.0%)	46 (41.1%)	.284
Race/ethnicity, n (%)			
African American/Black	2 (2.0%)	5 (4.5%)	.734
American Indian/Alaska Native	4 (4.0%)	2 (1.8%)	
Hispanic/Latino	3 (3.0%)	3 (2.7%)	
White	89 (89.0%)	99 (88.4%)	
Other	2 (2.0%)	3 (2.7%)	
Laboratory values at admission, mean (SD)			
Bilirubin, mg/dL	22.35 (7.81)	13.74 (7.46)	<.001
PT, s	16.70 (2.35)	21.73 (4.94)	<.001
Creatinine, mg/dL	1.36 (1.59)	2.43 (5.77)	.072
Sodium, mmol/L	131.43 (6.51)	133.06 (6.25)	.064
Albumin, g/dL	2.88 (0.52)	2.66 (0.60)	.007
Severity scores, mean (SD)			
MDF	43.97 (16.44)	58.49 (27.22)	<.001
MELD 3.0	37.33 (6.24)	36.60 (6.84)	.424
Clinical features, n (%)			
Hepatic encephalopathy	24 (24.0%)	46 (41.1%)	.013
Sepsis	11 (11.0%)	18 (16.1%)	.383
Insurance type, n (%)			
Private	79 (79.0%)	87 (77.7%)	.019
Medicaid	8 (8.0%)	20 (17.9%)	
Medicare	13 (13.0%)	5 (4.5%)	
Steroid treatment, n (%)			
Prednisolone	86 (86.0%)	85 (75.9%)	.092
Prednisone	14 (14.0%)	27 (24.1%)	

SD, standard deviation.

Evaluation of candidate clustering solutions demonstrated that a 2-cluster model (k = 2) provided the best separation (average silhouette width 0.59), indicating a well-defined and parsimonious structure (Supplementary Figure 2). The silhouette plot confirmed good within-cluster cohesion and between-cluster separation (Supplementary Figure 3). A dominance index reflecting the relative contributions of bilirubin and PT showed high concordance with the k-means classification (92% agreement; κ = 0.84).

Based on K-means clustering of the standardized bilirubin and PT components of the MDF, 100 patients (47.2%) were classified as bilirubin-driven and 112 (52.8%) as PT-driven. Cluster separation was confirmed through 2-dimensional visualization of the standardized inputs, showing differentiation along the PT and bilirubin axes (Supplementary Figure 1). The bilirubin-driven group was slightly younger than the PT-driven group, although this difference was not statistically significant (44.9 vs 47.4 years; *P* = .099). HE was less frequent in the bilirubin-driven group (24.0% vs 41.1%; *P* = .013). The bilirubin-driven group had a significantly lower median MDF score (44.0 vs 58.5; *P* < .001), while median MELD 3.0 scores were comparable between groups (37.3 vs 36.6; *P* = .42).

IPTW achieved adequate covariate balance (Supplementary Figure 4). In IPTW-weighted KM analysis, 90-day mortality was higher in the bilirubin-driven group than in the PT-driven group (35.5% [95% CI, 21.2%–49.9%] vs 20.2% [95% CI, 11.5%–28.8%]) (Figure). In IPTW-weighted Cox PH, the bilirubin-driven group had a significantly higher hazard of 90-day mortality (HR, 2.06; 95% CI, 1.05–4.04). The PH assumption was not violated for the bilirubin-driven vs PT-driven comparison (*P* = .08). In the Fine-Gray competing risks model, which accounted for LT as a competing event, the subdistribution hazard for 90-day mortality remained elevated in the bilirubin-driven group (sHR, 2.11; 95% CI, 1.03–4.35), consistent with the estimate from the Cox PH model. The main results of the regression models are summarized in Table 2.

**Figure:**
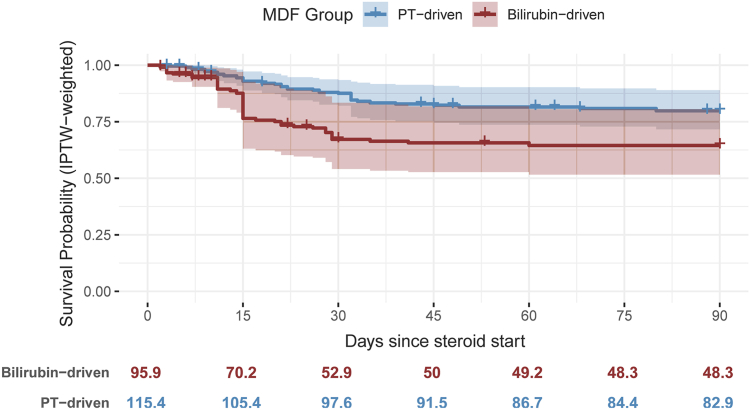
IPTW-weighted Kaplan-Meier survival curves. Kaplan-Meier curves comparing 90-day survival between the bilirubin-driven and PT-driven phenotypes after inverse probability of treatment weighting (IPTW). Shaded areas represent 95% confidence intervals.

**Table 2:** Association Between Bilirubin-Driven vs PT-Driven Phenotypes and 90-Day Mortality: IPTW-Weighted Cox Proportional Hazard and Fine-Gray Model Results

MDF phenotype	Cox PH model, HR (95% CI)	Fine-Gray model, sHR (95% CI)
Bilirubin-driven (ref: PT-driven)	2.06 (1.05–4.04)	2.11 (1.03–4.35)

IPTW was estimated using the following covariates: MDF score, age, sex, race, insurance type, intensive care unit stay, gastrointestinal bleeding, hepatic encephalopathy, sepsis, baseline AST, albumin, sodium, creatinine, and corticosteroid regimen.

Modeling the dominance index as a continuous variable yielded directionally consistent but statistically nonsignificant associations with 90-day mortality in both the Cox PH model (HR, 1.09; 95% CI, 0.86–1.37, *P* = .47) and the Fine-Gray competing risks model (sHR, 1.08; 95% CI, 0.86–1.36, *P* = .50), suggesting the binary k-means classification may better capture the threshold effect of bilirubin dominance. K-means and dominance-based binary groupings showed substantial agreement (Cohen's κ = 0.84, *P* < .001).

Among 205 patients surviving at least 7 days, Lille scores did not differ significantly between the bilirubin-driven and PT-driven groups (mean 0.256 vs 0.292; *P* = .17 by *t*-test, *P* = .10 by Wilcoxon test), though the bilirubin-driven group showed a numerical trend toward higher scores. Among 202 patients with discharge data, steroids were discontinued at discharge at similar rates between the groups: 40 (41.7%) in the bilirubin-driven group and 49 (46.2%) in the PT-driven group (*P* = .54).

## Discussion

In this study, we applied K-means clustering to investigate the prognostic significance of the individual components of the MDF, comparing bilirubin-driven and PT-driven phenotypes in patients with severe AH receiving corticosteroid therapy. The bilirubin-driven group appeared to have more favorable baseline clinical characteristics, including lower MDF scores, a lower prevalence of HE, and, though statistically nonsignificant, younger age. Nonetheless, after IPTW weighting with relevant confounders, both Cox and Fine-Gray models showed that the bilirubin-driven phenotype was associated with a higher risk of 90-day mortality.

Our findings suggest that serum bilirubin may exert a greater influence on short-term mortality than PT when conditioned on MDF. This indicates that, among patients with the same overall MDF severity, bilirubin potentially contributes more prognostically meaningful information regarding short-term mortality than PT. This contrasts with the findings of a European study, which used the Lille model score at day 7 as the endpoint rather than directly assessing mortality.^13^ Because the Lille model incorporates baseline PT as its only PT-related variable, concluding that baseline PT predicts a higher Lille score is inherently limited; it reflects mathematical coupling rather than a true causal relationship.

The bilirubin-driven group showed numerically higher Lille scores in our study, though the difference was not statistically significant (*P* = .10), and this subgroup ultimately had better adjusted 90-day survival than the PT-driven group. This discordance suggests that the standard practice of stopping corticosteroids solely based on day-4 or day-7 Lille thresholds may not fully capture treatment response in bilirubin-dominant disease.

In a prior study examining histologic predictors of mortality in AH, hepatocellular injury showed no association with outcomes, whereas bilirubinostasis was strongly linked to increased mortality as well as increased risk of bacterial infections during hospitalization.^22^ It is hypothesized that bilirubinostasis reflects underlying hepatocellular dysfunction, which increases susceptibility to bacterial infection; in turn, bacterial products may further impair biliary secretion, perpetuating a vicious cycle. Also, in a study analyzing liver samples from patients transplanted for severe, medically refractory AH, prothrombin levels were similar between those with AH and individuals with alcohol-associated cirrhosis without AH. However, bilirubin levels were strikingly higher in AH compared to patients with cirrhosis alone, underscoring its role as a distinguishing marker.^23^ These observations align with our findings, reinforcing the notion that bilirubin might play a more predominant role in prognostication in severe AH.

Our study has some limitations. First, it is retrospective in design, which carries an inherent risk of selection bias and unmeasured confounders. Second, it was conducted at a single tertiary care center, potentially limiting the generalizability of our findings to other settings or patient populations. Second, although several prognostic models exist beyond MDF and MELD, such as the Glasgow Alcohol-associated Hepatitis Score^24^ and the Age-Bilirubin-INR-Creatinine score,^25^ we only focused on MDF; however, MDF remains one of the most widely used criteria in daily practice and clinical trials of AH,^26^, ^27^, ^28^, ^29^ which supports its relevance and justifies examining how its individual components contribute to prognosis. Lastly, we did not perform interaction analyses for steroid response or evaluate the incremental prognostic value over MELD via C-statistics or reclassification; thus, our data do not directly support differential treatment strategies and warrant further prospective studies.

Progress in the management of AH relies on 3 key strategies: evaluating novel therapies, early identification of patients unlikely to respond to corticosteroids, and timely selection of candidates who may benefit from early LT evaluation.^30^ We believe our findings may contribute to advancing these efforts by providing a basis for hypothesis generation.

## Conclusion

We characterized a bilirubin-driven phenotype in patients with severe AH that is associated with increased 90-day mortality. While these findings highlight the clinical heterogeneity of the disease, further research is required to determine whether identifying this phenotype can improve risk stratification or inform the timing of LT evaluation and listing.

## Supplementary Materials

Supplementary Material

Extended PDF
