# Supplementary material for: Bilirubin-Driven Phenotype Identifies Higher Mortality Risk in Severe Alcohol-Associated Hepatitis
Source: Gastro Hep Adv. 2026 Apr 24;5(7):100982. doi: 10.1016/j.gastha.2026.100982 (PMC13217471; doi:10.1016/j.gastha.2026.100982)

APPENDIX: Bilirubin-Driven Phenotype Identifies Higher Mortality Risk in Severe Alcohol-associated Hepatitis

Tewodros T. Ayele, Tomohiro Tanaka

CLUSTER VALIDATION AND ROBUSTNESS ANALYSES 2
SUPPLIMENTAL FIGURES 3

Cluster validation and robustness analyses

To evaluate the robustness and interpretability of the clustering approach, we performed additional validation analyses. We assessed candidate cluster solutions (k = 2-7) using average silhouette width, which quantifies within-cluster cohesion and between-cluster separation.

To examine stability and transparency of the classification, we derived a dominance index representing the relative contribution of bilirubin and prothrombin time to the Maddrey Discriminant Function (MDF). The index was calculated as the difference between standardized bilirubin and prothrombin time components (z-bilirubin component – z-PT component). Patients with values >0 were classified as bilirubin-dominant and those ≤0 as PT-dominant.

Concordance between the dominance index classification and k-means clustering assignments was evaluated using percent agreement and Cohen's kappa statistic. In sensitivity analyses, the dominance index was also modeled as a continuous variable to assess its association with mortality outcomes.

Figure S1. Two-dimensional plot of K-means clustering based on standardized bilirubin and PT components of Maddrey's Discriminant Function, demonstrating clear separation into bilirubin-driven and PT-driven phenotypes.

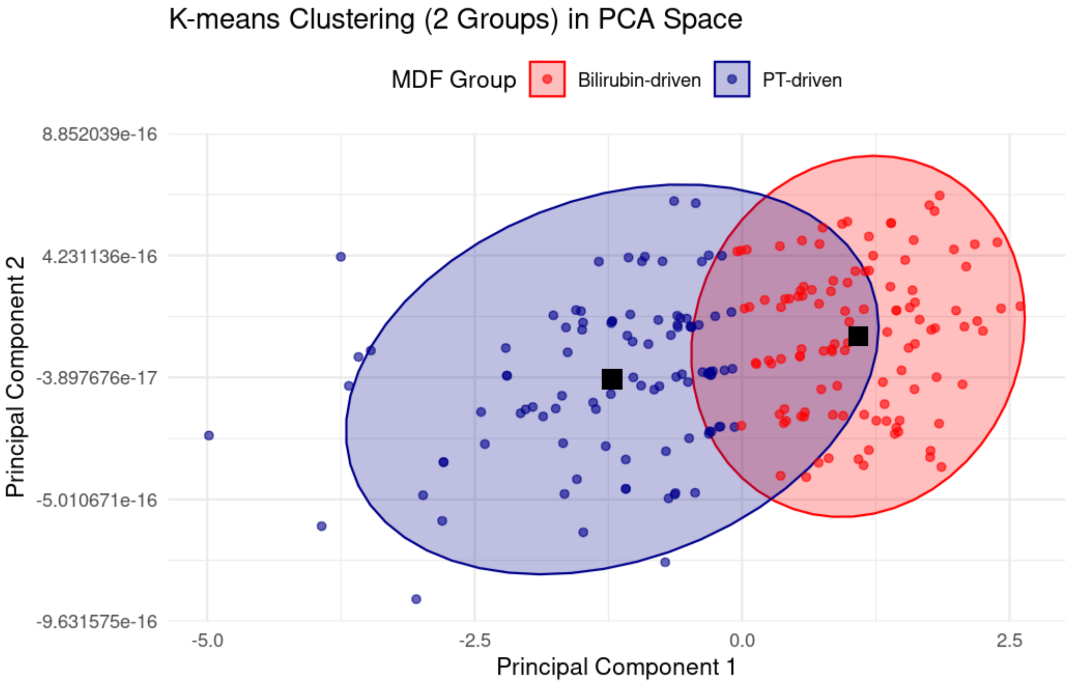

Figure S2. Average silhouette width across candidate clustering solutions (k = 2-7). The two-cluster solution demonstrated the highest silhouette width (0.59), indicating optimal separation and supporting a parsimonious clustering structure.

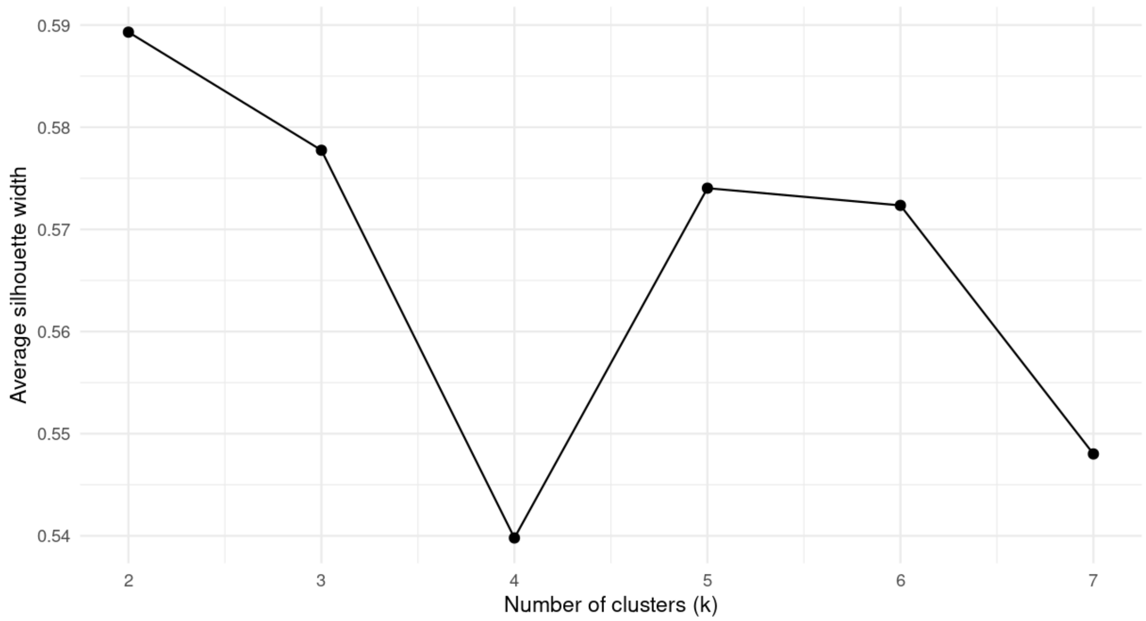

Figure S3. Silhouette plot for the two-cluster solution. Most observations demonstrate strong within-cluster cohesion and between-cluster separation, supporting the stability and interpretability of the two-cluster classification.

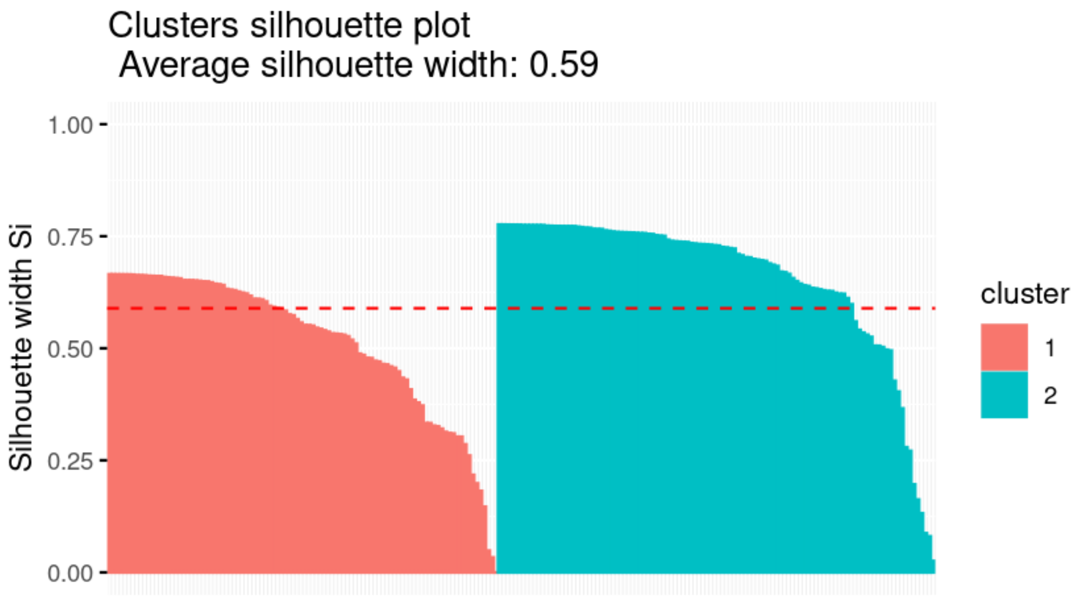

Figure S4. Covariate balance before and after inverse probability of treatment weighting (IPTW). Love plot showing absolute standardized mean differences for baseline covariates comparing the bilirubin-driven and PT-driven groups before (unadjusted) and after IPTW. After weighting, all covariates demonstrated improved balance, with standardized mean differences below the prespecified threshold.

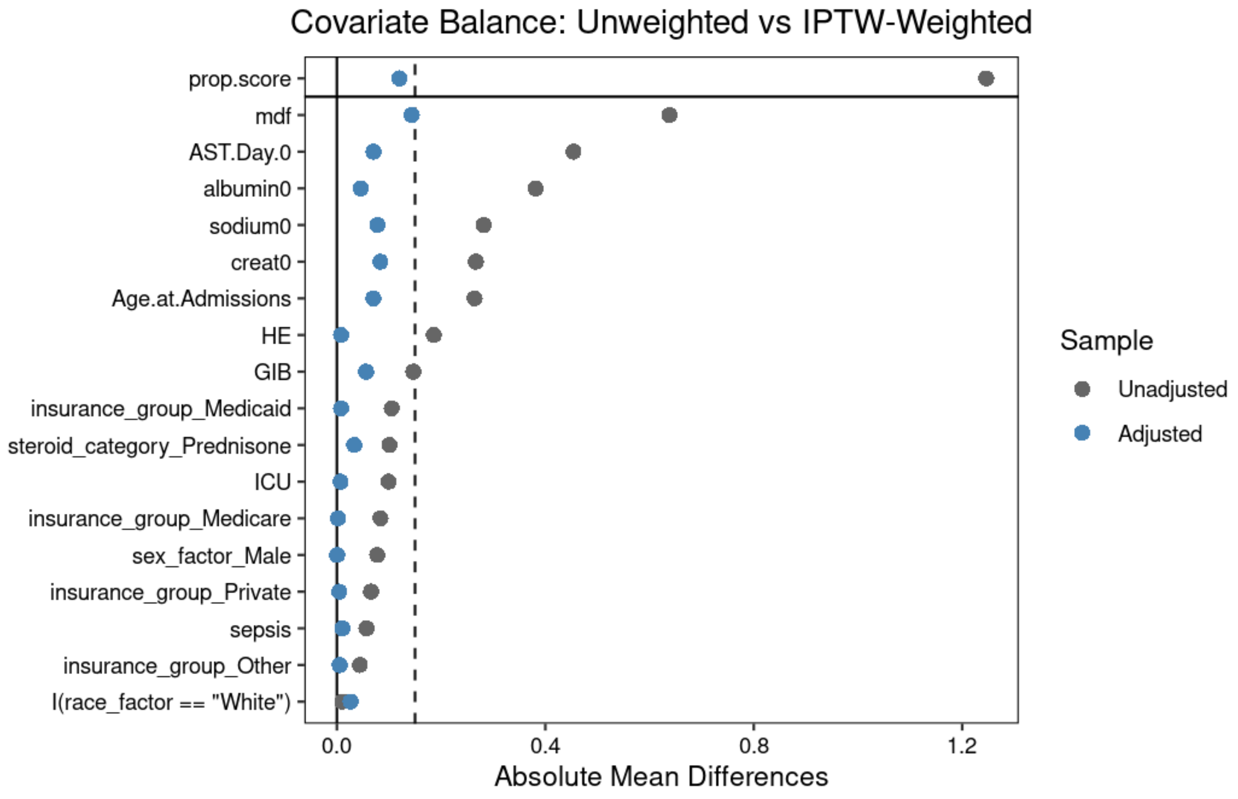

Supplement: Supplementary Material [file mmc1.pdf]
